# Supplementary material for: Identification of single nucleotide variants using position-specific error estimation in deep sequencing data
Source: BMC Med Genomics. 2019 Aug 2;12:115. doi: 10.1186/s12920-019-0557-9 (PMC6679440; doi:10.1186/s12920-019-0557-9)
Supplement: Supplementary file 1 — Supplementary Methods. Details of the MutPlat pipeline. (DOCX 15 kb) [file 12920_2019_557_MOESM1_ESM.docx]

**Supplementary Methods**

**The MutPlat pipeline**

Given a set of bam files, corresponding to N tumour samples and a germline from the same patient (bam_T1_ bam_T2_, …, bam_TN_ bam_GL_), the MutPlat pipeline implemented in this work consists of the following basic steps:

1. Run Mutect2 (GATK version 3.6) with default parameters on each tumour/germline pair (bam_T1_ bam_GL_ bam_T2_/bam_GL_, …, bam_TN_bam_GL_). This generates N vcf files (vcf_T1_ vcf_T2_, …, vcf_TN_.
2. From the N vcf files above, generate a single vcf file, vcf_T_ which overlays all positions present in the individual vcf files. As an example if vcf_T1 _= {pos_1_, pos_2_, pos_3_} and vcf_T2 _= {pos_2_, pos_4_} then vcf_T _= {pos_1_, pos_2_, pos_3_, pos_4_}. All positions in the individual vcf files are included, independently of e.g. coverage and filter flag assigned by Mutect2.
3. Run Platypus (version 0.8.1) jointly on (bam_T1_ bam_T2_, …, bam_TN_ bam_GL_) using the vcf file, vcf_T_, from step 2 as prior. More specifically we set the options ‘--source=vcf_T_’ and ‘--getVariantsFromBam=1’.

The outcome is a multi-sample vcf file that is subsequently filtered as described in the Methods section to extract germline and somatic SNVs.
