# Supplementary figures and images for: Identification of single nucleotide variants using position-specific error estimation in deep sequencing data
Source: BMC Med Genomics. 2019 Aug 2;12:115. doi: 10.1186/s12920-019-0557-9 (PMC6679440; doi:10.1186/s12920-019-0557-9)

## Slide 1
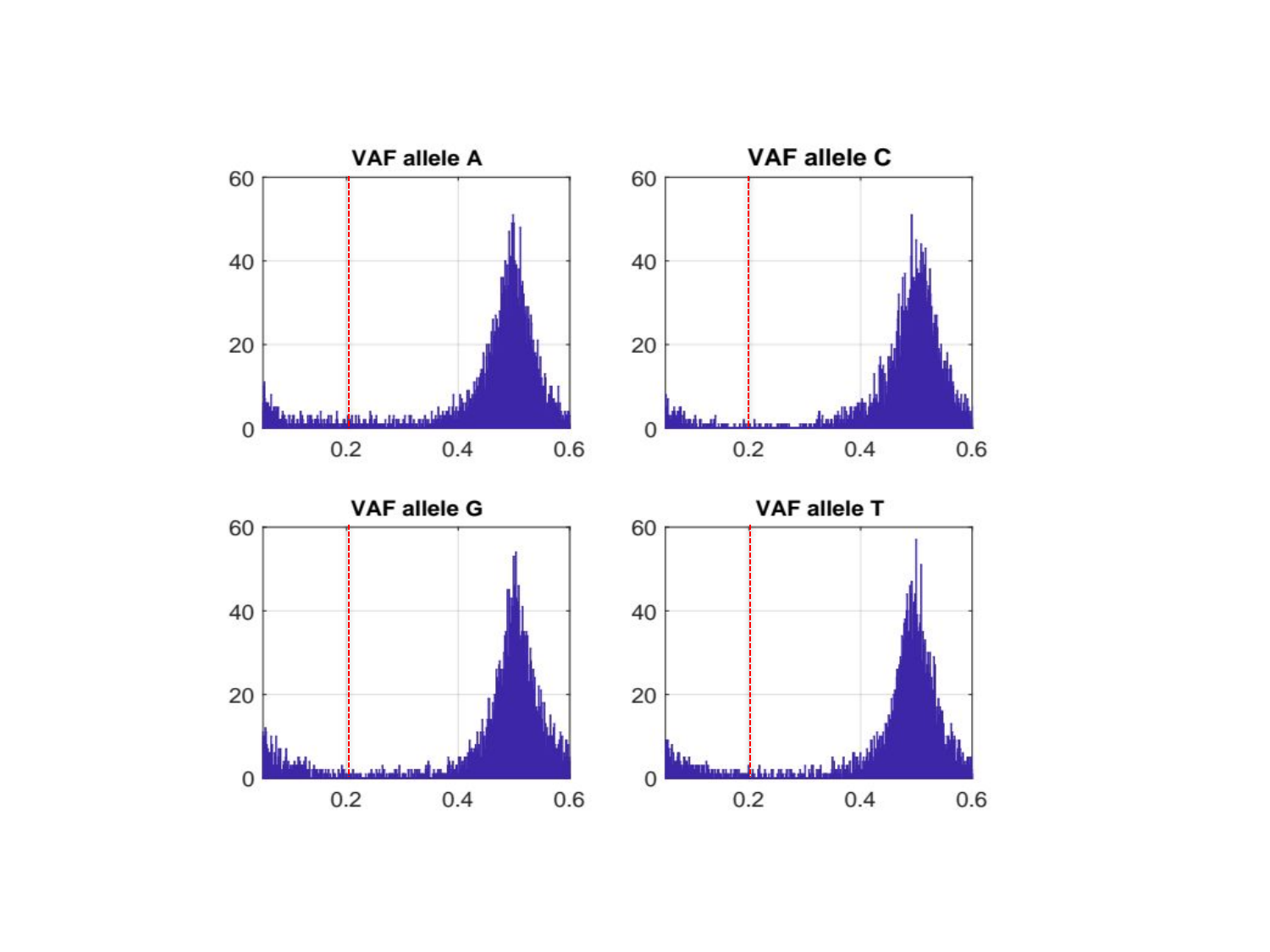

Supplement: Supplementary file 2 — Figure S1. Variant allele frequency (VAF) distributions for the A, T, C, G nucleotides as calculated from 30 randomly chosen normal samples across our custom AmpliSeq panel. Only VAFs < 60% are displayed. The red lines mark VAF = 20%. (PPTX 278 kb) [file 12920_2019_557_MOESM2_ESM.pptx]

## Slide 1
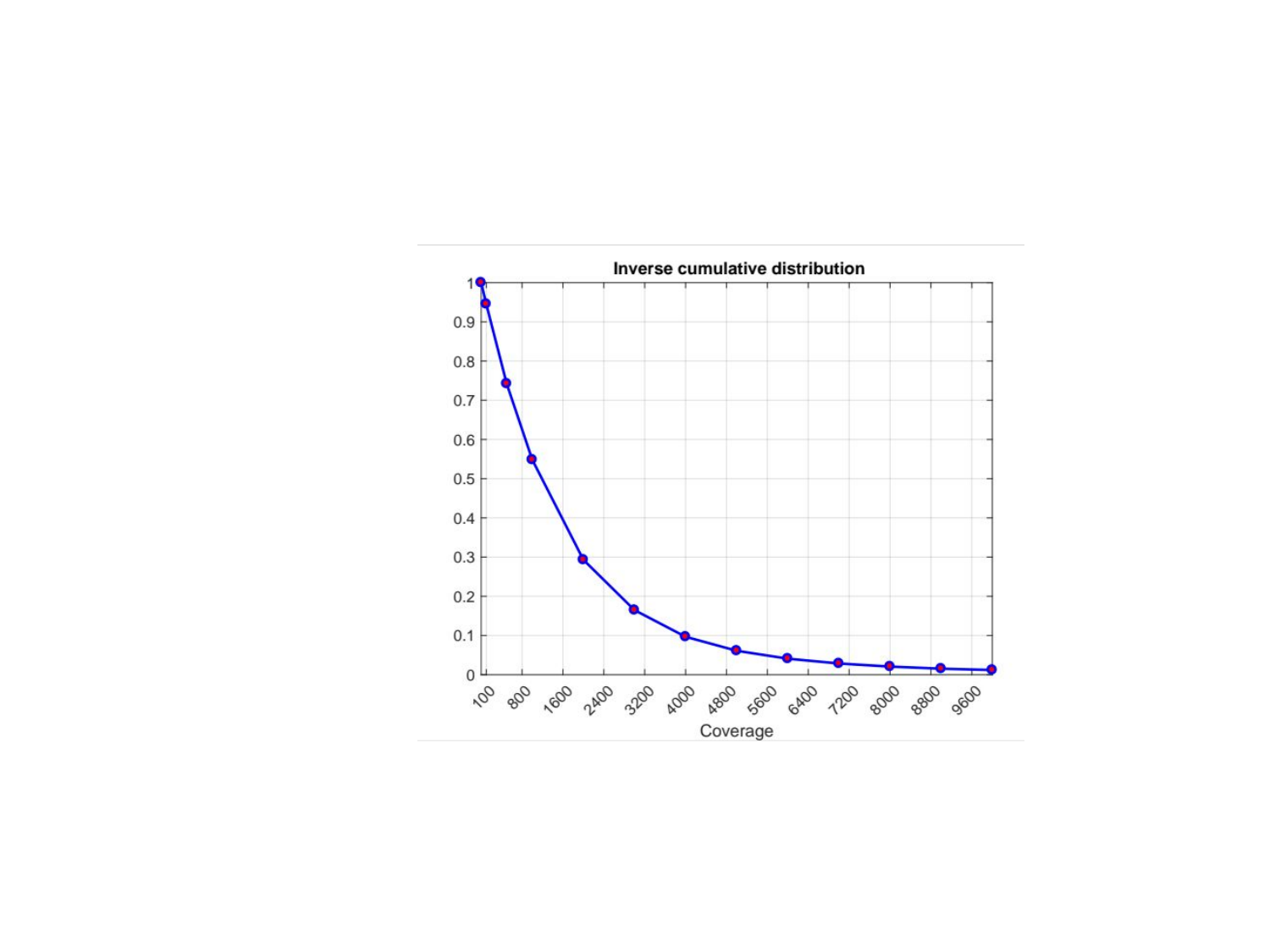

Supplement: Supplementary file 3 — Figure S2. Fraction of sites in a normal sample sequenced at a given coverage or more across our custom AmpliSeq panel. The values are calculated over 30 randomly selected samples. (PPTX 59 kb) [file 12920_2019_557_MOESM3_ESM.pptx]

## Slide 1
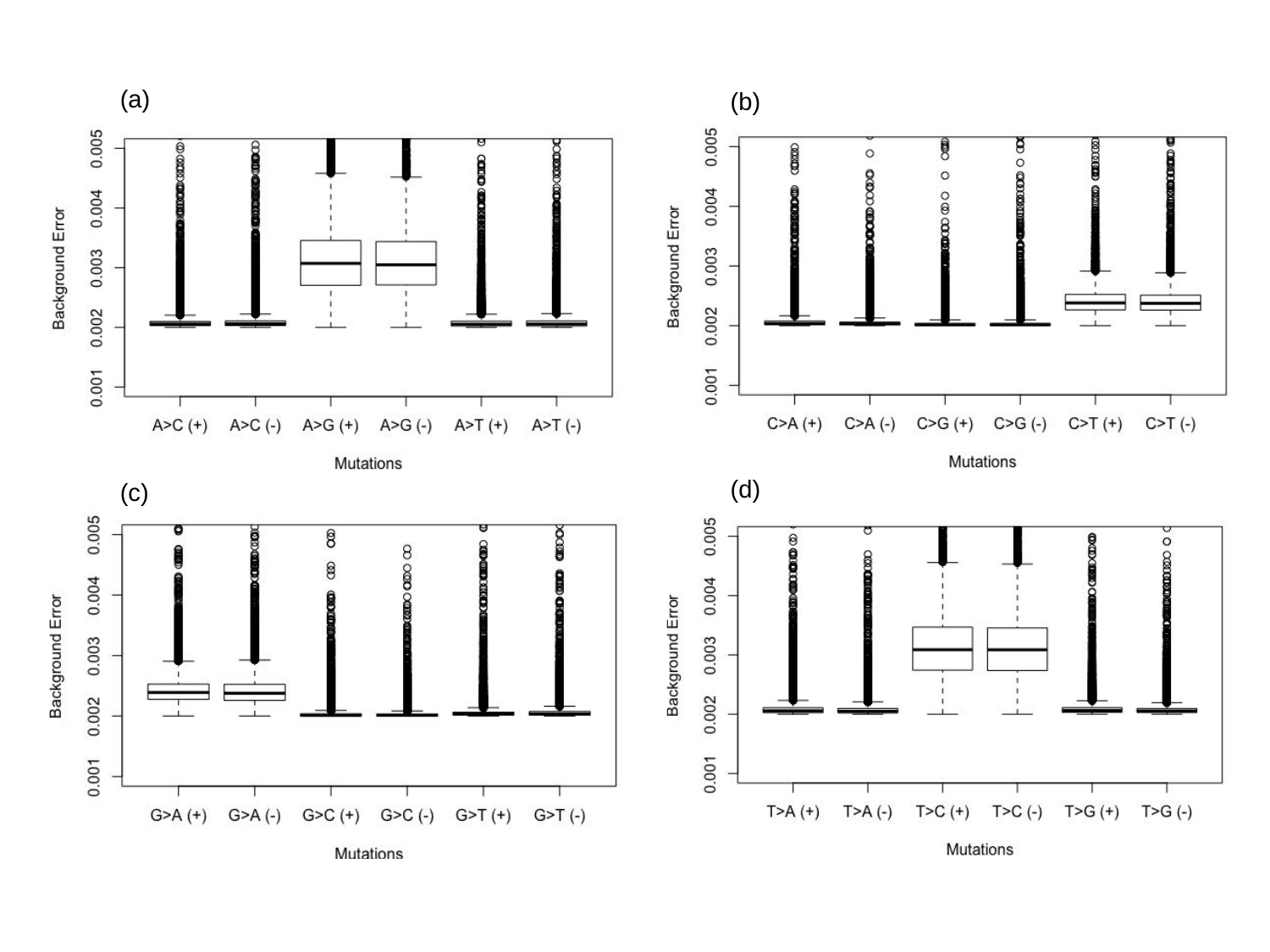

(a)
(b)
(d)
(c)

Supplement: Supplementary file 4 — Figure S3. Distributions of background error values by mutation type. Panels (a), (b), (c) and (d) refers to mutations from reference allele A, C, G and T respectively. Mutations are split by alternative allele and strand, (+) and (−). Note the higher error values for A > G (T > C) and C > T (G > A) mutations. Plots are bound to error values of 0.005 on the y-axis for visual clarity. (PPTX 201 kb) [file 12920_2019_557_MOESM4_ESM.pptx]

## Slide 1
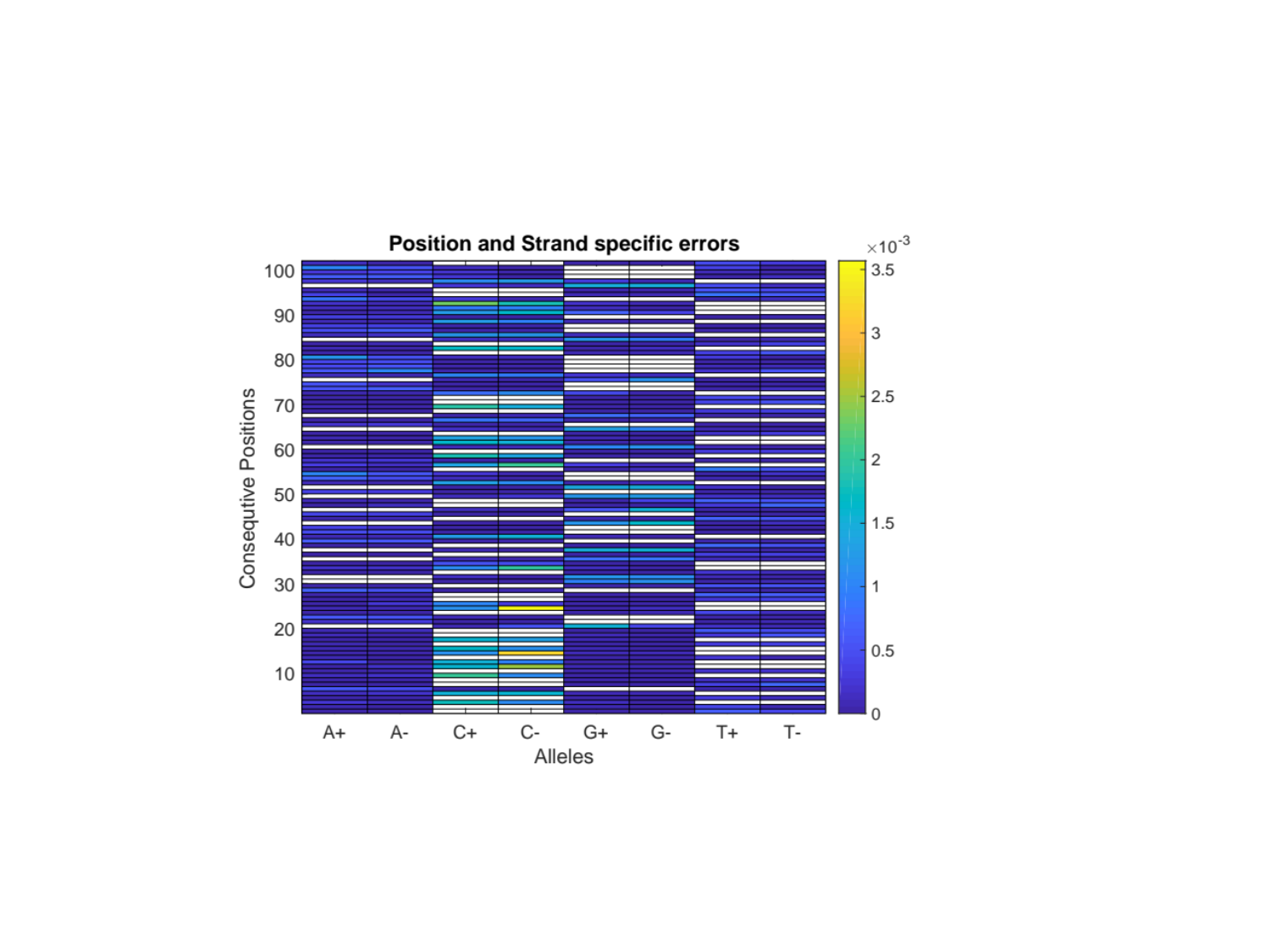

Supplement: Supplementary file 5 — Figure S4. Position-specific, allele-specific and strand-specific frequency of alternative alleles in 100 consecutive positions in the AR gene. (PPTX 116 kb) [file 12920_2019_557_MOESM5_ESM.pptx]

## Slide 1
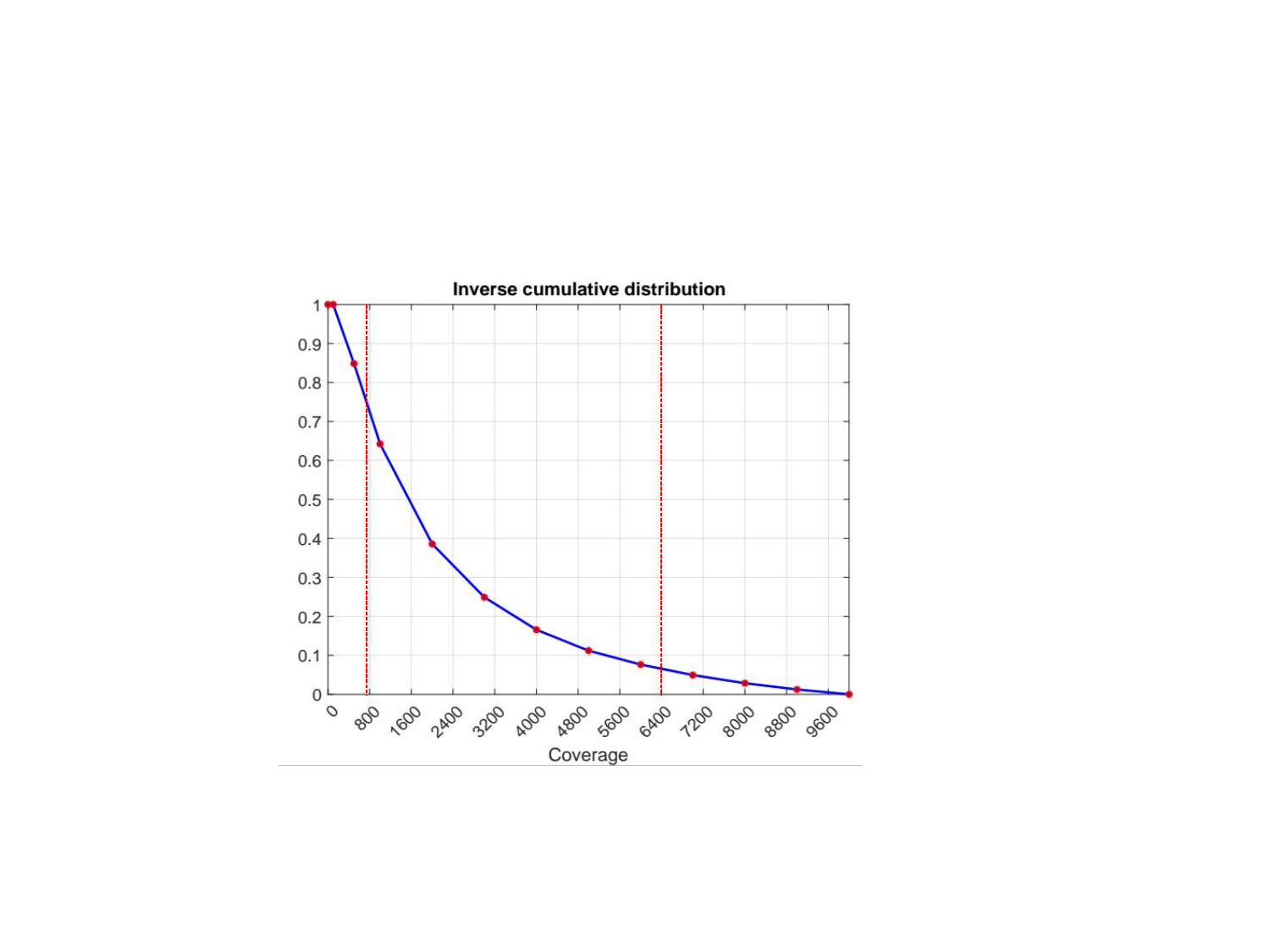

Supplement: Supplementary file 6 — Figure S5. Fraction of sites in our custom AmpliSeq panel sequenced at a given coverage or more. The values are calculated over 30 randomly selected ctDNA samples. Note that positions with depth of coverage less than 200 are not considered for calculating the total number of positions. The red lines represent the upper and lower bounds of coverage used in the synthetic variant test. (PPTX 64 kb) [file 12920_2019_557_MOESM6_ESM.pptx]
